# Unveiling the Invisible: Inferior STEMI With an Absent Right Coronary Artery Rescued by PCI to the Left Circumflex Artery

**DOI:** 10.1155/cric/8887458

**Published:** 2026-04-09

**Authors:** Jehan Dad Khan, Omar Bajmmal, Ibrahim Antoun

**Affiliations:** ^1^ Department of Cardiology, Kettering General Hospital, Kettering, UK, nhs.uk; ^2^ Division of Cardiovascular Sciences, College of Life Sciences, University of Leiceste, Leicester, UK, le.ac.uk

**Keywords:** absent coronary artery, acute coronary syndrome, coronary angiogram, myocardial infarction, right coronary artery

## Abstract

Coronary artery anomalies, though uncommon, can pose significant diagnostic and therapeutic challenges, especially during acute coronary syndromes (ACS). We present a rare case of a 57‐year‐old male who presented with classical symptoms of inferior ST‐segment elevation myocardial infarction (STEMI). Initial coronary angiography failed to identify the right coronary artery (RCA), eventually revealing an absent RCA ostium. The superdominant left circumflex artery (LCX) was found to be entirely occluded, supplying the territory typically serviced by the absent RCA. Successful primary percutaneous coronary intervention (PCI) was performed using a drug‐eluting stent (DES), restoring TIMI‐III flow and resolving symptoms promptly. Further imaging with contrast‐enhanced computed tomography coronary angiography (CTCA) confirmed the congenital absence of the RCA, emphasising the critical role of advanced imaging in diagnosing coronary anomalies. Our case underscores the importance of considering rare congenital anomalies in acute cardiac presentations. It highlights the pivotal role of rapid recognition, targeted imaging and intervention in such anomalies to ensure optimal patient outcomes. It advocates a multidisciplinary approach, integrating cardiologists, radiologists and interventional specialists to enhance clinical decision‐making and management effectiveness in similar complex scenarios.

## 1. Introduction

Cardiovascular disease is a global healthcare challenge, especially in the developing world [1]. Congenital anomalous variations of the coronary arteries are rare and often remain undetected [2]. A few anomalies that are frequently seen in daily practice include the high‐origin coronary artery, multiple ostia, aberrant origin from the opposite/noncoronary valsalva sinus, single coronary artery, anomalous left coronary artery from the pulmonary artery (ALCAPA), duplications of the left anterior descending artery, coronary fistulas and extracardiac termination [3]. Presentation is usually linked to various clinical scenarios when imaging studies are carried out, ranging from benign cases to acute myocardial infarction (MI) and sudden cardiac death. One particularly rare and clinically significant anomaly is the absent right coronary artery (RCA), with an approximate incidence of 0.01%–0.07% in the general population [4], which is often asymptomatic but can present with symptoms of ischemia and other life‐threatening complications when associated with cardiovascular events, such as acute coronary syndrome (ACS).

Anomalous coronaries can be detected with invasive coronary angiography. However, the diagnosis is increasingly supported by computed tomography coronary angiography (CTCA), which is noninvasive and provides detailed information on coronary anatomy. Coronary artery anomalies are found in 0.2%–1.3% of patients undergoing coronary angiography and 0.3% of an autopsy series. In the settings of absent RCA, the left coronary artery (LCA) often assumes the role of supplying the inferior wall [5]. The importance of early detection and management cannot be overstated, as it can alter the therapeutic approaches, including the decision to perform primary percutaneous coronary intervention (PPCI) in patients with ST‐segment elevation myocardial infarction (STEMI).

The case discussed here highlights a patient who presented with an inferior STEMI with absent RCA and superdominant left circumflex, found to be the primary vessel supplying the inferior myocardial territory and was treated with successful PCI to the left circumflex artery (LCX) with one drug‐eluting stent (DES).

## 2. Case Presentation

**Figure 1:**
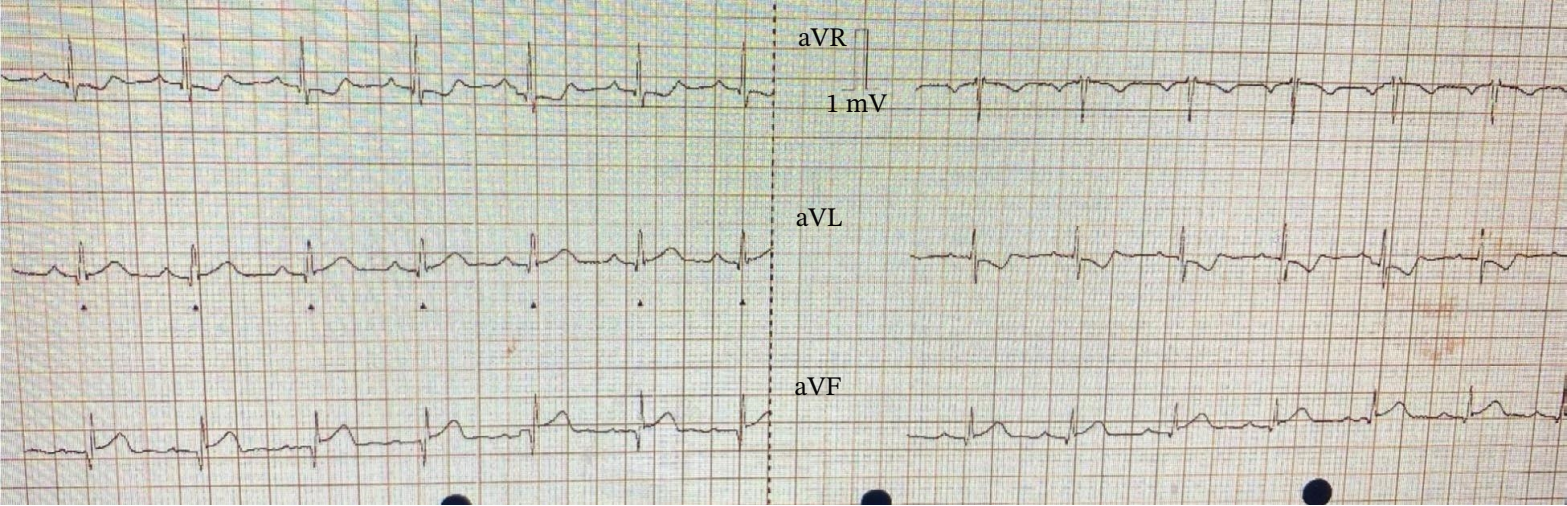
12 leads electrocardiogram on admission showing inferior ST elevation in keeping with ST‐elevation myocardial infarction.

A 57‐year‐old male with a family history of premature coronary artery disease was brought in by ambulance with central chest pain for 2 hours, which was radiating to the left arm/jaw and was associated with cold sweating and nausea. On examination, his pulse was regular and 75 beats per minute; on auscultation, his chest was clear, with normal heart sounds. An electrocardiogram (ECG) performed by the ambulance crew had ST‐segment elevation in Leads II, III and aVF, along with reciprocal changes in line with an inferior STEMI (Figure 1). She was taken to the cardiac catheter laboratory for PPCI.

**Figure 3:**
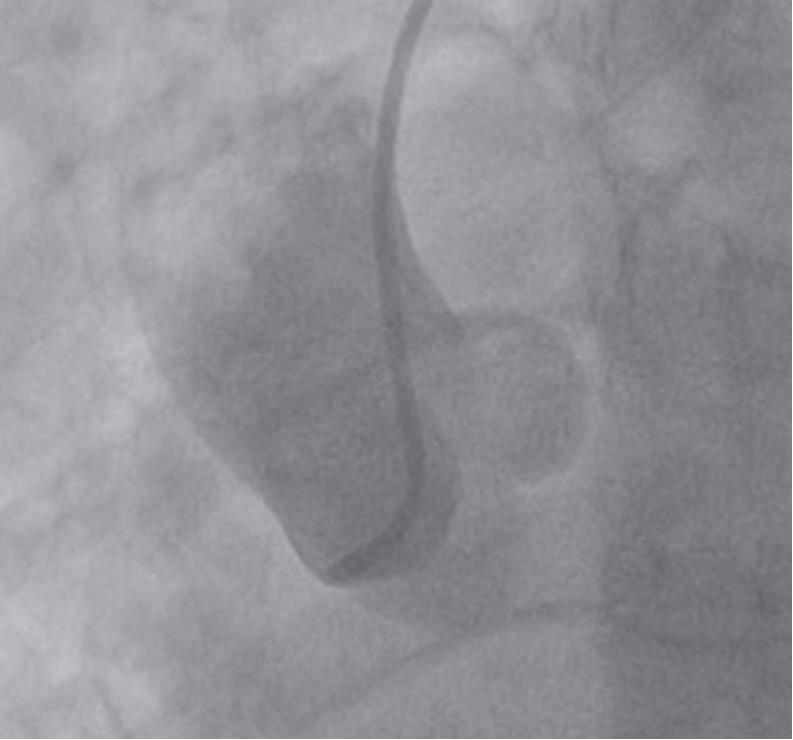
Coronary angiogram demonstrating aortic root injection, confirming the absence of the right coronary artery ostium at the expected anatomical location.

The patient underwent coronary angiography via a right femoral approach after right radial access could not be obtained. Attempts were made to engage the RCA with Judkins right and Amplatz left catheters, including a contrast injection in the right aortic cusp to engage the ostium of the RCA, with no success in visualising the ostium of RCA at its expected anatomical location (Figure 2). The left main stem (LMS) was then engaged using a Judkins left catheter, followed by a contrast injection, showing a dominant left coronary system with an occluded LCX (Figure 3a). A BMW guidewire was parked in the distal LCX, and the lesion was dilated with a balloon RYUREI 1.25 × 10 and then a RYUREI 2.0 × 15. Another contrast injection after balloon inflation showed that the superdominant LCX was supplying the inferior wall (RCA territory) (Figure 3b). The lesion was then stented with an ULTIMATE Tansei 3.0 × 24 mm DES with excellent angiographic results and achieved TIMI‐III flow (Figure 3c). The patient was admitted to the coronary care unit (CCU) and was started on aspirin and ticagrelor with close monitoring for any signs of further ischemia or complications. A repeat ECG postprocedure revealed resolution of the ST‐segment elevation and T‐wave inversions in the inferior leads, with downtrending troponins in the following 24 h. The patient remained stable throughout his recovery.

Figure 2Coronary angiographic images illustrating the intervention process: **(2A)** Diagnostic angiogram demonstrating a completely occluded dominant left circumflex artery (green arrow). **(2B)** Balloon angioplasty of the left circumflex artery lesion with visible balloon inflation (blue arrow). **(2C)** Post‐intervention angiogram showing successful reperfusion and restored TIMI‐III flow in the left circumflex artery after deployment of a drug‐eluting stent (DES) (red arrow).

**Figure:**
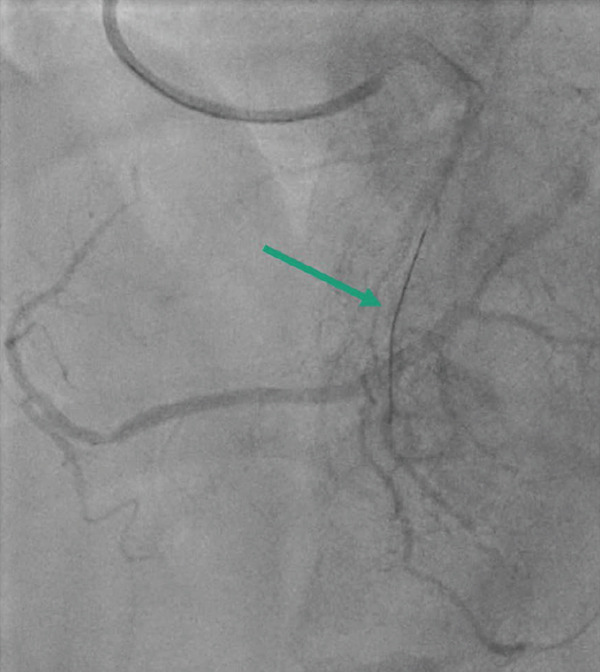
(a)

**Figure:**
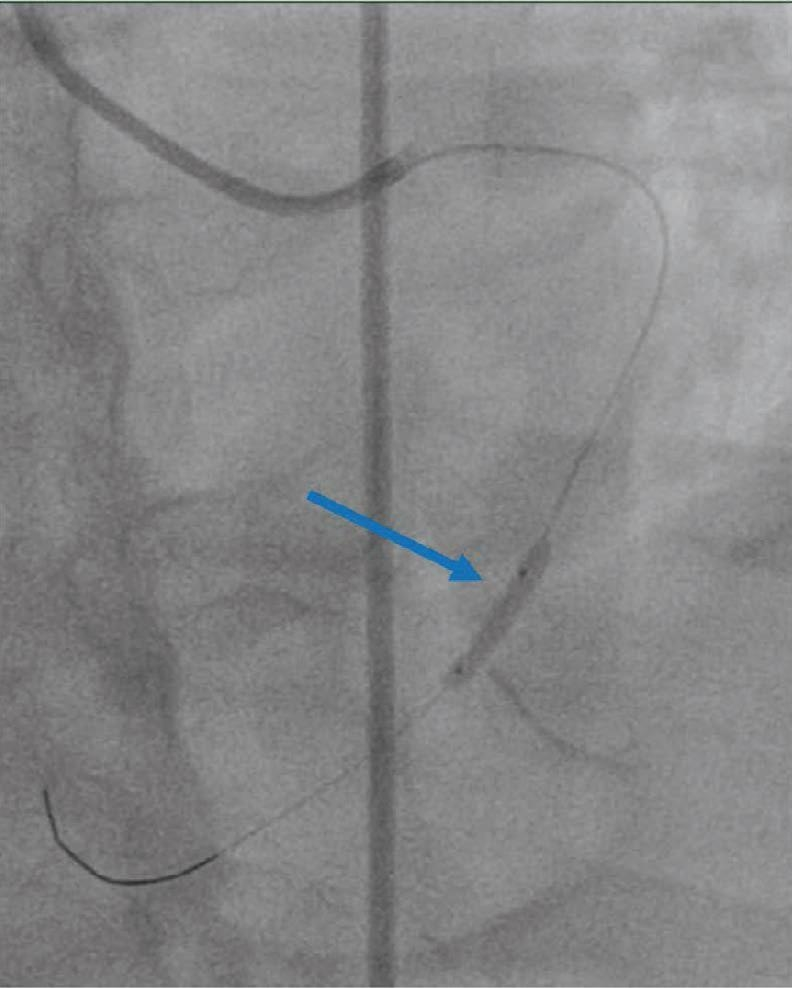
(b)

**Figure:**
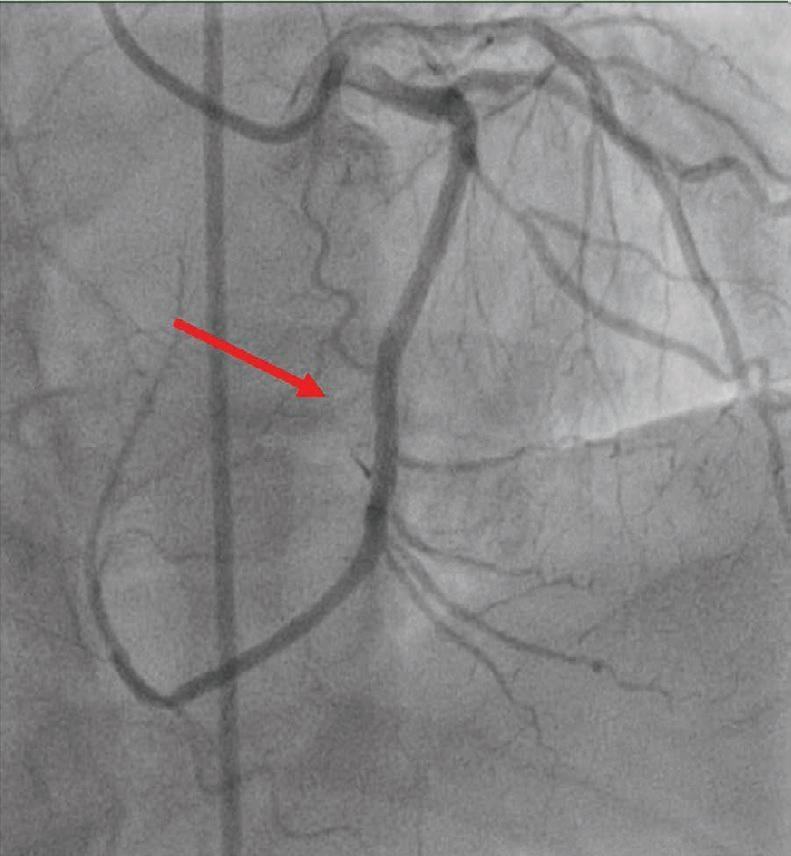
(c)

**Figure 4:**
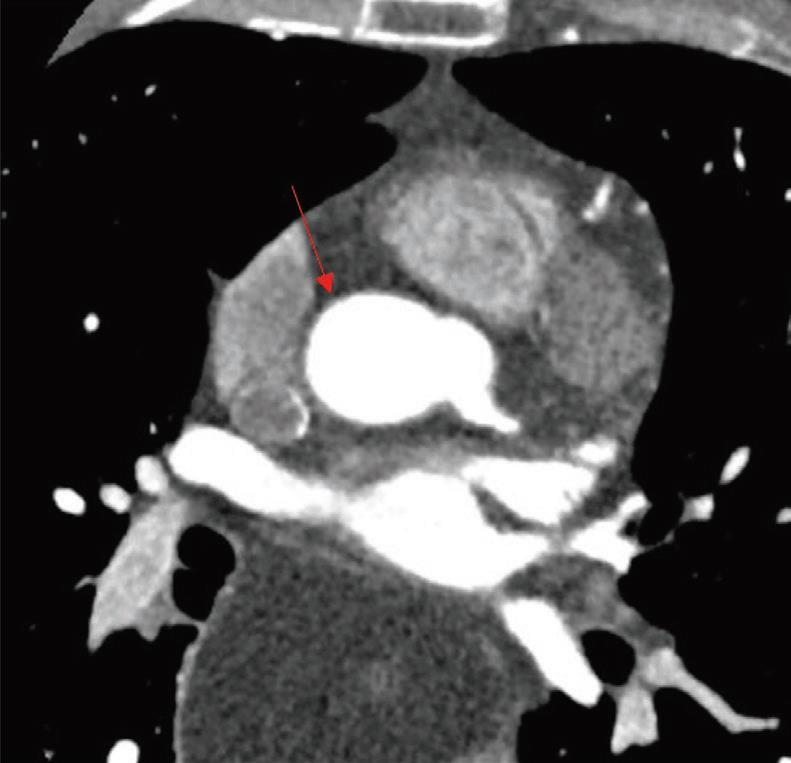
Contrast‐enhanced computed tomography coronary angiography (CTCA) axial view confirming the congenital absence of the right coronary artery ostium (red arrow) from the right coronary sinus.

To confirm the absence of RCA ostium, further imaging with CTCA was done after, which confirmed the angiographic finding of absent RCA ostium (Figure 4). A transthoracic echocardiogram demonstrated a structurally normal heart and preserved biventricular size and systolic function.

The patient was discharged 72 h after the procedure, with recommendations for lifestyle modifications, blood pressure and lipid management and regular follow‐up with cardiology. At 1‐year follow‐up, the patient remains stable and is discharged to primary care.

## 3. Discussion

The present case underscores the critical necessity of heightened clinical suspicion when a coronary ostium is difficult to engage during angiography, particularly in the acute setting of STEMI. Although conventional coronary angiography remains the cornerstone for immediate intervention, our case illustrates the substantial complementary role of noninvasive imaging modalities, such as CTCA, in confirming anomalous coronary anatomy and guiding postintervention clinical decision‐making.

Prior studies have shown that congenital coronary anomalies are associated with increased risks during interventional procedures due to atypical vessel trajectories and unforeseen anatomy [6]. Although rare, the congenital absence of the RCA poses specific diagnostic and therapeutic challenges, particularly in the context of acute MI, where prompt reperfusion is critical [7]. This case reiterates that the dominance and compensatory enlargement of contralateral vessels, such as the LCX, are common adaptations in response to congenital absences, potentially masking critical anatomical deviations until acute clinical events precipitate their detection [8].

Our experience aligns with previously reported cases in which DES deployment has proven effective, affirming the suitability of standard PCI techniques for managing anomalous coronary presentations [9]. However, the risk of procedural complications or technical difficulties remains significant, underlining the importance of expertise and meticulous preprocedural imaging assessment. Furthermore, advocating a multidisciplinary team approach is vital for comprehensively managing these complex presentations, from initial diagnosis through therapeutic intervention to subsequent patient management.

Future research focusing on systematic screening methods that incorporate routine CTCA in cases with atypical angiographic findings or procedural challenges may enhance early detection rates of coronary anomalies, thereby optimising therapeutic outcomes and potentially averting catastrophic cardiovascular events. Additionally, integrating artificial intelligence (AI) in image analysis and clinical decision‐making platforms may further enhance the diagnostic accuracy, procedural planning and overall management of patients with rare coronary anomalies, potentially transforming outcomes in acute cardiac care.

## 4. Conclusion

This case emphasises considering coronary anomalies, like absent RCA, when engaging a coronary ostium is difficult, especially in ACS. Advanced imaging such as CTCA is crucial for diagnosis and guiding treatment. Early detection and proper management can improve outcomes in these complex cases.

**Nomenclature**
STEMI: ST‐elevation myocardial infarction
ACS: acute coronary syndrome
CTCA: computed tomography coronary angiogram
PCI: percutaneous coronary intervention
DES: drug‐eluting stent
LCX: left circumflex artery
RCA: right coronary artery
ECG: electrocardiogram

## Author Contributions

Ibrahim Antoun: conceptualization; data curation; writing—original draft. Jehan Dad Khan: writing—review and editing. Omar Bajmmal: writing—review and editing.

## Funding

No funding was received for this manuscript.

## Consent

The patient gave written informed consent to publish this report in accordance with the journal′s patient consent policy and for their clinical details along with any identifying images to be published in this study.

## Conflicts of Interest

The authors declare no conflicts of interest.

## Data Availability

This manuscript does not report data generation or analysis.
